# *Vpar_1595* encodes malate–lactate transhydrogenase: The first step in lactate metabolism within the genus *Veillonella*

**DOI:** 10.1080/29933935.2026.2641986

**Published:** 2026-03-14

**Authors:** Maxwell Calvin Guillaume, Filipe Branco dos Santos

**Affiliations:** a Swammerdam Institute for Life Sciences, Molecular Microbial Physiology Group, University of Amsterdam, Amsterdam, The Netherlands

**Keywords:** Malate–lactate transhydrogenase, *Veillonella*, *Vpar_1595*, MLTH, mouth

## Abstract

The genus *Veillonella* plays a pivotal role in human oral microbiota and biofilm formation. The role of these bacteria is to act as intermediaries, which they do partly by metabolizing L-lactate. Interestingly, their first step in lactate metabolism is catalyzed by malate‒lactate transhydrogenase (MLTH), an enzyme so far unique to the genus *Veillonella* and one whose sequence is yet unknown. Here, we identified the elusive MLTH sequence from a strain of *Veillonella parvula* and its corresponding gene *Vpar_1595* based primarily on enzyme purification and LC‒MS/MS analysis, with the purified MLTH matching the theoretical *Vpar_1595* protein spectrum with a MASCOT score of 4657. In addition, we show that both the molecular weight of 40 kDa and the predicted amino acid composition of *Vpar_1595* correspond to the MLTH described in previous reports. We then perform a phylogenetic analysis and identify 88 putative MLTH-encoding genes within the genus *Veillonella*, showing that it indeed forms a distinct clade. This work provides the first molecular identification of the *Veillonella* malate–lactate transhydrogenase, establishing a genetic basis for a key metabolic step in oral biofilm formation. These findings open the door to study MLTH's evolutionary origins and its potential as a target for modulating oral microbial interactions.

## Graphical abstract

**Figure:**
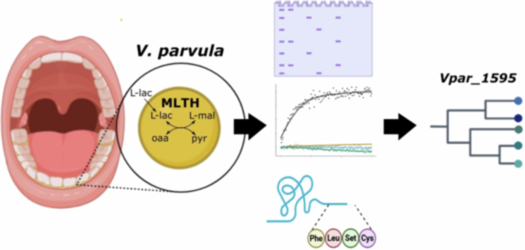

## Introduction

The human oral cavity is an environment of dizzying diversity. Among the cavities and multifarious surfaces, with the ever present overflow of proteinaceous saliva, arise local gradients of various ecological factors, such as temperature, pH, redox potential, and nutrients.^1^ In order to survive in this dynamic habitat, first colonizers of polymicrobial communities take a specialist approach and adhere to their preferred ecological niche.^2^ From there, these communities almost exclusively form biofilms and play an integral role between the homeostasis of oral health and disease. However, there is one member, in particular, that plays a pivotal role in the development of these communities: the genus *Veillonella.*^3^

*Veillonella* is the third most abundant genus in the oral cavity and consists of obligate anaerobic Gram-negative cocci. Known as a “bridging taxa,” *Veillonella* species provide an interface between the initial colonizers and the later arrivals.^4^^,^^5^ In addition to providing a structural and interdependent scaffold for the microbial community, they also act as a metabolic intermediary. A primary carbon source for *Veillonella* is the fermentative end-product lactate that is produced by early colonizers, such as streptococci or lactobacilli.^6^ Lactate is one of the strongest organic acids produced to a significant degree by the oral microbiota and helps induce demineralization of hard dental tissues. Thus, its consumption by *Veillonella* may play a role in ameliorating these effects.^3^^,^^7^ Furthermore, lactate is summarily metabolized to propionate and acetate, both of which have been linked to human health.^8-10^

Interestingly, the first step in *Veillonella* lactate metabolism is catalyzed by malate‒lactate transhydrogenase (MLTH), an enzyme thought to be unique to their genus. First identified and studied in the mid-20th century, this enzyme catalyzes a bidirectional reaction: L-lactate + oxaloacetate ⇔ L-malate + pyruvate (EC 1.1.99.7). Notably, it was determined that this enzyme does not use mobile NAD^+^/NADH cofactors, but rather catalyzes this reaction in a single step using a covalently bound NAD moiety to transfer the electrons in a “ping-pong” manner.^11-13^ Assays showed that the reaction could only be carried out if both reactants from the same side were present.^11^ Furthermore, previous reports determined that this enzyme forms a homodimer and measured the subunit molecular weight as 40,000 ± 1000 Da.^12^^,^^14^ The amino acid composition was also measured, but not all proteinogenic amino acids were accounted for due to the limitations of the day.^12^^,^^14^

Unfortunately, it appears that interest in this enzyme dwindled down before DNA or protein sequencing became common practice; thus, the sequence for an MLTH is yet unknown. However, with the resurgence of interest in the genus *Veillonella* due to its impact on human health and the central position that MLTH takes in *Veillonella* metabolism, we have isolated the MLTH from a strain of *Veillonella parvula* and identified its sequence using LC-MS/MS. We then heterologously expressed and purified this enzyme in *Escherichia coli* and confirmed its activity *in vitro*. Moreover, we show that previously reported characteristics of MLTH, such as amino acid composition and subunit molecular weight, are corroborated by our findings. We then perform a phylogenetic analysis to identify putative MLTH genes within the genus *Veillonella*. With the putative MLTH sequences, we then compare them to related sequences and show that they form their own distinct clade.

## Materials and methods

### *Veillonella parvula* cultivation and lysate preparation

*Veillonella parvula* DSM 2008 was cultivated as described previously.^11^ In essence, the culture was grown anaerobically for 48 h after inoculation at 30 °C in a 1 L vessel with a culture volume of 500 mL. The medium employed consisted of 1% (m/v) yeast extract, 1% (m/v) tryptone, and 2% (m/v) sodium L-lactate as the carbon source.

The culture was then centrifuged at 14,000 rpm for 25 min at 4 °C and then resuspended in chilled cell lysate buffer (50 mM Tris, 1 mg/mL lysozyme, 2.5 mg/mL DNAse, 2.5 mg/mL RNAse, pH 7.6) to a final concentration of 30% (m/v). The resuspended culture was then sonicated on ice for 5 min in 30 s on/off intervals. The crude cell extract was subsequently centrifuged for 45 min at 14,000 rpm. The supernatant was then transferred to a Spectra/Por® dialysis membrane with a pore size of 10 kDa and placed in 3 L of 20 mM Tris, pH 7.6, at 4 °C, and left stirring overnight.

### MLTH assay

The assay used to detect MLTH activity is similar to the direct assay described by Allen.^14^ For each measurement, we added 0.05 mL of 0.5 M Tris, pH 7.6, 0.1 mL of 0.1 M sodium pyruvate, 0.05 mL of 0.2 M DL-malic acid, 0.04 mL of H_2_O, and 0.01 mL of fractionated cell lysate or purified enzyme. Unless otherwise stated, a background measurement was taken as blank before adding the cell lysate or enzyme. Absorbance was measured at 258 nm to monitor oxaloacetate formation^11^ using a spectrophotometer (Lightwave II, Biochrom).

### FPLC & SDS‒PAGE isolation

The dialyzed cell lysate was first fractionated via an ÄKTA FPLC system (GE Healthcare) using a HiQtrap HP 5 mL column. Approximately, 20 mL of the *V. parvula* cell lysate was loaded onto the column and washed with 50 mL of 20 mM Tris, pH 7.6. The elution buffer was added to the column using a linear gradient over the course of 12.5 min and consisted of 1 M NaCl, 20 mM Tris, pH 7.6. The total flow rate was held constant at 4 mL/min throughout both the loading and elution phases. Protein content was monitored inline by measuring the UV absorbance at 280 nm. Eluent was collected in 4 mL fractions, which were directly placed on ice. Fractions that showed higher protein content upon elution phase compared to protein content during the loading phase were assayed for MLTH activity in the manner described above. The fractions that showed positive activity for MLTH were selected and pooled for further purification.

The positive assay fractions were first concentrated from 4 mL to 0.2 mL using an Amicon® Ultra Centrifugal Filter, 10 kDa MWCO, and centrifuged at 3000 rpm for 40 min. A Superdex 200 column was placed on the FPLC and 100 µL of concentrate was loaded using 20 mM Tris, 100 mM NaCl, pH 7.6 buffer, with a flow rate of 0.3 mL/min. Fractions were collected in 0.5 mL portions. The protein content was measured as before, and fractions with protein content higher than the baseline content were assayed.

Positive assay fractions from both the HiTrap Q HP and Superdex200 runs were then run on an SDS‒PAGE gel. A total of 15 µL from each fraction were loaded into separate wells of an ExpressPlus™ PAGE Gel, 10 × 8, 8%–16% from Genscript and run according to the manufacturer's instructions. The ladder used was PageRuler™ Prestained Protein Ladder, 10–180 kDa. The gel was then stained using PageBlue™ Protein Staining Solution from Thermo Fisher.

### Mass spectrometry MLTH identification

The sample handling, software analysis, and equipment used for the identification of the MLTH gene via mass spectrometry is almost identical to enzyme identification procedures described previously.^15^ In short, the designated gel band from the SDS‒PAGE gel was excised and subjected to an in-gel trypsin digestion method.^16^ Eluate was collected after a 30-min extraction with 10 mM NH_4_HCO_3_ and then freeze-dried, reconstituted in 20 μL of 50% acetonitrile and 2% formic acid, and stored at −20 °C overnight.

About 2 μL of stored sample was dried using a speedvac for 5 min and resuspended in 7 μL of 2% acetonitrile and 0.1% trifluoroacetic acid. About 5 μL of the resuspended sample was loaded and separated with an eluent flow of 300 nL min^−1^ on an Acclaim PepMap100 (C18 75 μM 25 cm Dionex, Thermo Scientific) analytical column combined with an Acclaim PepMap100 precolumn (C18 100 μM 2 cm Dionex, Thermo Scientific) using a 30 min gradient of 0%–50% ACN and 0.1% formic acid. The LC‒MS/MS system employed was an AmaZon Speed Iontrap MS/MS with a CaptiveSpray ion source (Bruker) inline with an EASY-nLC II (Proxeon, Thermo Scientific).

The data processing and searching parameters with Data Analysis software (Bruker) and Mascot (version 2.5.1) were done exactly the same as previous^15^ with the exception that the search database was derived from the *V. parvula* DSM 2008, complete genome (GenBank: CP001820.1).

### Amino acid composition comparison

The amino acid composition for MLTH was previously reported.^12^ The composition was measured by hydrolyzing MLTH with HCL at two different time points, namely, 15 h and 23 h. A python script was written to calculate the amino acid compositions for all protein sequences in the same fasta file that was used to create the search database in the previous section. Each protein's amino acid composition was compared to the reported MLTH amino acid composition at the 15-h time point and then sorted based on the sum absolute difference in percentage for each amino acid. Owing to limited technology at the time,^12^ did not report measurements for the amino acids asparagine and glutamine. However, these amino acids were still accounted for when calculating percentages for the other amino acids, but were not included when summing the absolute difference for each protein compared to the MLTH.

### Molecular cloning

The newly identified *mlth* gene was synthesized from Integrated DNA Technologies (Europe) with an *N*-terminal His-tag and flanking NdeI/XhoI restriction sites, which was provided to us in the plasmid pUC-*mlth*. This plasmid was digested with NdeI/XhoI, and the resulting *mlth* fragment was ligated into a pET28a backbone fragment that was digested with the same restriction enzymes, yielding the pET28a-*mlth* plasmid. Strains and plasmids used in this study are listed in Table 1.

**Table 1.** List of strains and plasmids used in this study.

Strain/plasmid	Description	Reference
*V. parvula* DSM 2008	*V. parvula* strain used in this study	[17]
XL1 blue	*E. coli* competent cells for routine cloning	[18]
BL21DE3	*E. coli* expression host	[19]
pUC-*mlth*	KanR; T-vector containing the His-tagged *mlth* from Genscript	This study
pET28a	KanR; routine expression vector with *lac* operator	[20]
pET28a-*mlth*	KanR; expression vector containing His-tagged *mlth*	This study

### MLTH expression and purification

The pET28a-*mlth* plasmid was transformed into *E. coli* BL21DE3 cells. A single colony was inoculated into 600 mL of LB medium supplemented with 50 µg/mL kanamycin and grown to an OD of 0.8. IPTG was then added to a final concentration of 1 mM, and the culture was left mixing overnight at room temperature. After 16 h, the cell lysate was extracted and prepared for protein purification via FPLC as described previously. After dialysis, the total protein content was measured using the BCA assay to calculate the proper sample volume such that the column would not be overloaded.

To purify the His-tagged MLTH from the cell lysate, 10 mL of sample was loaded onto a HisTrap™ HP 5 mL column with 50 mL of loading buffer (20 mM Tris, 150 mM NaCl, 20 mM imidazole, pH 8.0) with a flow rate of 4 mL/min. A linear gradient was used to switch from 0% to 100% elution buffer (20 mM Tris, 150 mM NaCl, 500 mM imidazole, pH 8.0) over the course of 20 min. Eluate was collected in fractions and assayed for MLTH activity. Active fractions were combined, and the final concentration of protein was determined to be 6.6 µg/µL via a BCA assay.

A fraction of the combined eluate was run on SDS–PAGE as described previously to determine the quality of purification, with the exception of using the PageRuler™ Plus Prestained protein ladder (Thermo Fisher).

### Phylogenetic analysis

The putative MLTH sequences were obtained by performing a protein BLAST^21^ of the sequence for *Vpar_1595* against the nonredundant protein database with default parameters but limiting the taxonomy criterion to the order *Veillonellales*. We then filtered the results with conservative criteria of 90% query coverage and 70% identity, resulting in 88 sequences (Supplemental File 1).

Representative sequences were obtained for domains *AllD* (Accession: COG2055) and *Ldh_2* (Accession: pfam02615) from the NCBI Conserved Protein Domain Family database (https://www.ncbi.nlm.nih.gov/Structure/cdd/cdd.shtml, 24 September 2024), resulting in 463 and 322 sequences, respectively (Supplemental File 2). The reviewed enzyme sequences were obtained by performing a PSI-BLAST with default parameters and *Vpar_1595* as the input sequence in the SwissProt database. Two iterations were run, resulting in 46 hits, after which no new sequences were added, as none were below the default *E* value threshold of 0.05 (Supplemental File 3). All BLASTs were performed with the BLOSUM62 substitution matrix and default parameters.

All non-MLTH sequences in the multiple sequence alignment of canonical LDHs and MDHs were gathered from UniProt (16 October 2024). LDH (EC:1.1.1.27), MDH NAD+ (EC:1.1.1.37), and MDH NADP+ (EC:1.1.1.82) sequences were obtained by searching their corresponding EC numbers and selecting only the reviewed entries. This resulted in 311 LDH, 593 MDH NAD+, and 10 MDH NADP+ sequences (Supplemental File 4).

All multiple sequence alignments (MSA) were performed using the MAFFT (version 7) algorithm using the MAFFT online tool (https://www.genome.jp/tools-bin/mafft).^22^^,^^23^ The strategy employed was FFT-NS-i, and all other parameters were set to the default settings. The phylogenetic tree was constructed by importing the resulting MSA into TreeViewer (v2.2.0)^24^ and constructing a neighbor-joining tree with 1000 bootstrap replicates and default parameters, except without allowing negative branch lengths.

## Results

### Identification of MLTH from *V. parvula*

Although MLTH activity has been described in *V. parvula*, the gene responsible for this activity was never identified. In order to identify the MLTH gene, we purified proteins from *V. parvula* cell lysate and used a spectrophotometric assay to isolate enzymatic activity.

The cell lysate was first fractionated using anion exchange (here termed as Q fractions). Nine Q fractions contained protein according to UV absorbance, of which only two showed MLTH activity, Q18 and Q19 (Supplemental Figure 1A). Fraction Q18 was then concentrated and separated further on a size exclusion column. After this second round of fractionating (termed as S fractions), four fractions showed MLTH activity, S25–S28 (Supplemental Figure 1B).

**Figure 1.**
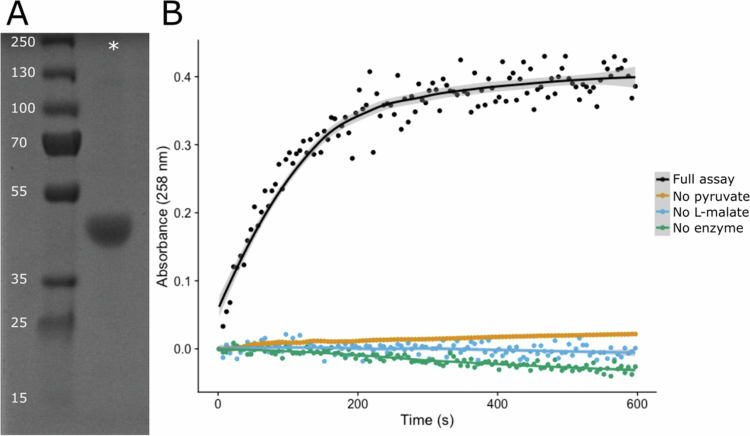
Characterizing purified *Vpar_1595* enzyme expressed in an *E. coli* host. (A) SDS‒PAGE of the purified protein. The numbers next to the ladder indicate the molecular weight (kDa) of the adjacent band. The * symbol indicates the well where the purified protein was loaded. (B) Direct assay of MLTH activity by measuring oxaloacetate formation via absorbance at 258 nm using the purified protein. Black: full assay with enzyme, pyruvate, and malate (full assay); orange: partial control assay with only enzyme and malate (no pyruvate); blue: partial control assay with only pyruvate and malate (no enzyme); green: partial control assay with only enzyme and pyruvate (no L-malate).

Subsequently, all positive fractions, both Q and S, were run on an SDS‒PAGE gel to determine purity (Supplemental Figure 1C). According to previous literature, the MLTH enzyme is a homodimer with a subunit molecular weight of 40,000 ± 1000 Da.^14^ Therefore, the band around 40 kDa from the S26 column was excised from the gel, digested, and analyzed by LC‒MS/MS. A short list of candidate proteins derived from a *V. parvula* genome was obtained, with the top hit, *Vpar_1595*, having a vastly higher protein coverage score than the next noncontaminant highest hit (4657 vs 64, Supplemental Table 1).

Moreover, previous research measured the amino acid composition of the MLTH enzyme.^12^ We calculated the amino acid compositions from sequences for all coding regions in the *V. parvula* genomes and compared them to the reported MLTH amino acid composition. Once again, *Vpar_1595* was the top candidate with the closest matching amino acid composition (Supplemental Table 2).

The sequence encoding *Vpar_1595* (Supplemental Table 3) was then synthesized with an *N*-terminal His-tag, placed in an inducible expression vector, and was heterologously expressed in *E. coli.* The resulting enzyme was then purified to a high degree using a HisTag column and verified on a gel, with a band at approximately 40 kDa (Figure 1A). The purified enzyme was then assayed with different variations of the MLTH assay, where only some or all of the substrates are present and/or the enzyme is present (Figure 1B). Note that this assay does not contain any mobile electron carriers, such as NAD^+^/NADH or NADP^+^/NADPH, and that activity is monitored by measuring oxaloacetate formation at 258 nm. The only variation of the assay that showed activity was the full assay, where both substrates, pyruvate and L-malate, and the enzyme are present. The slight negative slope of the controls with pyruvate (blue, green) is most likely due to interference of pyruvate, which partially absorbs light at 258 nm.^14^ Together, these results show that the purified protein from *Vpar_1595* has the same molecular weight and enzymatic activity as was previously reported for MLTH.^11^

#### Phylogenetic analysis

**Figure 2.**
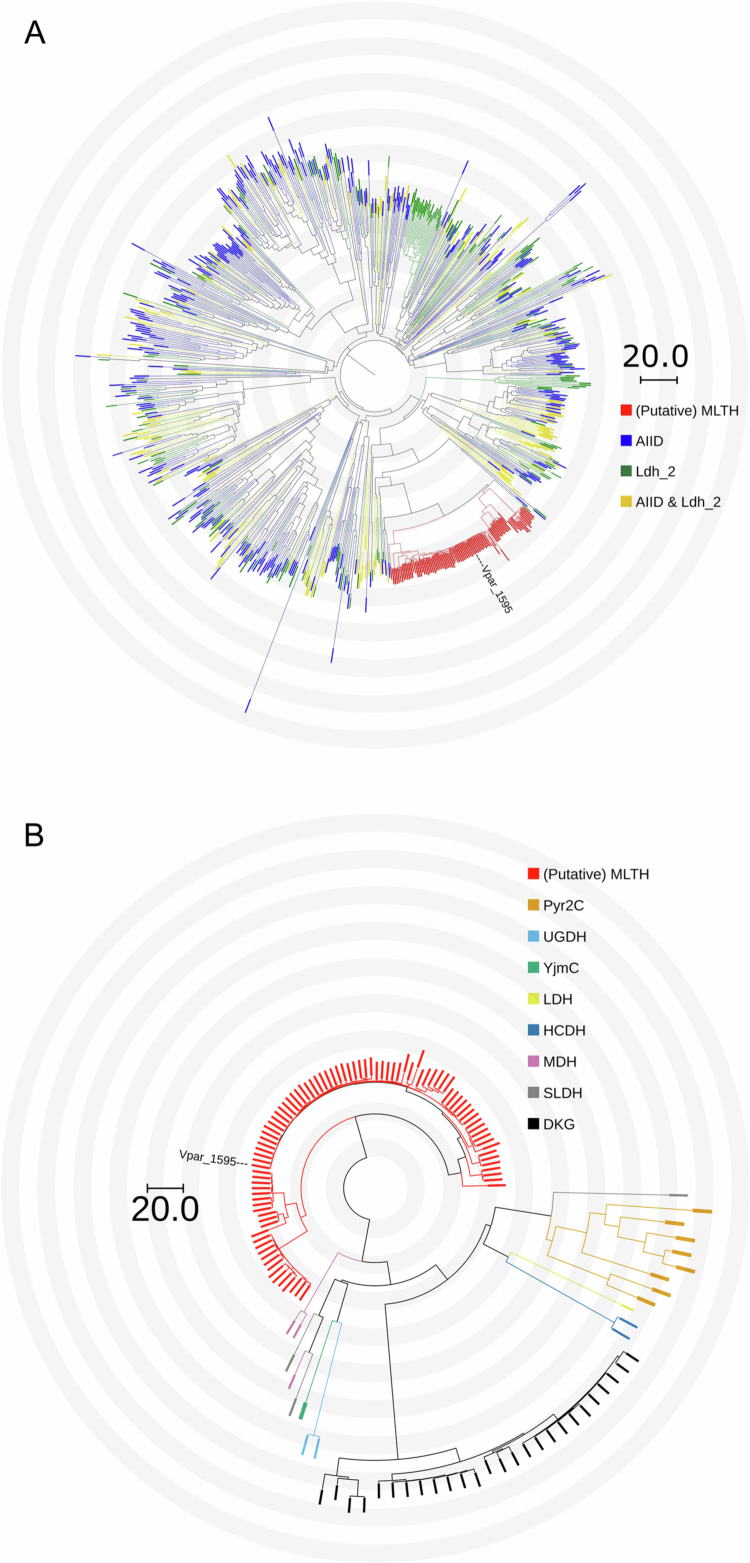
Phylogenetic trees comparing the putative MLTH sequences to conserved domains and reviewed SwissProt sequences. The distance scale radiates from the center of each tree, with gray and white bands indicating a distance of 10, as denoted by the scale bar, which traverses two bands. The node corresponding to *Vpar_1595* is highlighted in both trees. (A) Representative sequences for two conserved domains, AIID and Ldh_2, compared to MLTH sequences. Note that yellow indicates sequences in both AIID and Ldh_2. (B) PSI-BLAST results from SwissProt compared to MLTH sequences. MLTH = malate‒lactate transhydrogenase, Pyr2C = delta(1)-pyrroline-2-carboxylate reductase, UGDH = ureidoglycolate dehydrogenase, YjmC = oxidoreductase YjmC, LDH = lactate dehydrogenase, HCDH = hydroxycarboxylate dehydrogenase, MDH = malate dehydrogenase, SLDH = sulfolactate dehydrogenase, DKG = 2,3-diketo-L-gulonate reductase.

Using the *Vpar_1595* sequence as input for BLAST, we identified 88 putative MLTH sequences in the order *Veillonellales* after filtering for query coverage and identity (see Material & methods). Two conserved domains were also detected, namely AIID and Ldh_2. Ldh_2 is composed of bacterial and archaeal malate/L-lactate dehydrogenases, and AIID consists of malate, lactate, and ureidoglycolate dehydrogenases. We, therefore, gathered representative sequences for both domains and compared them to the putative MLTHs to determine how they cluster among seemingly related sequences (Figure 2A).

Although the putative MLTHs are nested within some of the representative sequences, they form a near monophyletic clade with maximal support (1.0), with a single Ldh_2 sequence nesting within the MLTH clade. This tight clustering indicates that the MLTHs may have a unique sequence among their peers. However, this could also be due to the conservative criteria that we chose to determine the putative MLTHs. Additionally, most of the domain representative sequences have unknown functions, so it is unclear from this tree how MLTH activity relates to other enzymatic activities. We, therefore, did a PSI-BLAST on SwissProt to see how the MLTHs stack up against enzymes with known functions (Figure 2B). Unsurprisingly, the hits found were all dehydrogenases and reductases, though with some diversity in function. Most of these are also part of the AIID domain family. However, we once again observed tight clustering of the putative MLTHs among related proteins, forming a monophyletic clade with maximal support (1.0).

Given the nature of MLTH's enzymatic activity and its similarity to both malate dehydrogenase and lactate dehydrogenase activities, we compared the putative MLTH sequences to the canonical LDH and MDH sequences (Supplemental Figure 2). The MLTH sequences not only form a monophyletic clade with maximal support (1.0) but also are distant from a large majority of canonical sequences, suggesting evolutionary divergence.

## Discussion

We have provided strong evidence that the gene *Vpar_1595* encodes MLTH enzyme in *V. parvula*. Among the proteins identified via LC–MS after the purification of MLTH activity from *V. parvula* cell lysate, *Vpar_1595* was by far the most prominent candidate. Recombinant expression of *Vpar_1595* confirmed that it catalyzes the MLTH reaction, with properties consistent with those previously reported for this enzyme. Specifically, we showed that (i) the dissociated enzyme exhibits a subunit molecular weight of ~40 kDa, which is consistent with earlier measurements of MLTH; (ii) the predicted amino acid composition of *Vpar_1595* aligns most closely with the empirically determined composition of purified MLTH when compared against all other annotated proteins in the *V. parvula* proteome; and (iii) MLTH activity was observed from the *Vpar_1595* product only when both L-malate and pyruvate were present, which reflects the known stoichiometry of the MLTH reaction.^14^

We next performed an exploratory phylogenetic analysis of the MLTH enzyme. Previous work suggested that MLTH is unique to the order *Veillonellales,*^5^ so we limited our initial search for additional putative MLTHs to this order, which resulted in 88 hits. We used highly conservative criteria of >90% query coverage and >70% identity, which far exceeds the commonly used 30% identity threshold for inferring functional relatedness,^25^ since automatic annotations of MLTHs or MLTH-like enzymes are not currently possible due to their previously unknown sequence. Neighbor-joining was used as a distance-based, exploratory approach to assess broad clustering patterns among putative MLTH sequences and diverse representative sequences with related functions.

Within the *V. parvula* DSM 2008 genome, the *Vpar_1595* protein was originally misclassified with an NCBI functional annotation of “Malate/L-lactate dehydrogenase.” Although seemingly similar, we show that the putative MLTHs and the *Vpar_1595* protein are distinct from both canonical malate dehydrogenases and L-lactate dehydrogenases (Supplemental Figure 2). The distance from these canonical dehydrogenases also suggests distant evolutionary lineages. Furthermore, MLTH enzymatic activity is also distinct from either MDH or LDH functionality and instead catalyzes the combination of the two latter enzymatic reactions. As mentioned previously, MLTH does not require mobile NAD^+^/NADH or NADP^+^/NADPH as a cofactor for its enzymatic activity (Figure 1B), which is corroborated by the fact that its amino acid sequence does not contain the highly conserved GxGxxG/A motif in Rossmann-fold enzymes. Updating these annotations in databases, such as the expanded Human Oral Microbiome Database,^26^ would facilitate a top-down strategy to identify additional MLTHs, possibly outside of the order *Veillonellales*, and allow for a deeper phylogenetic analysis.

For a broader phylogenetic framework, the putative MLTH-clade nests within representative sequences of both the *AIID* and *Ldh_2* domain families but remains distinct and tightly clustered, forming a near monophyletic clade with maximal support (Figure 2A). Although, this tight clustering is likely a consequence of a combination of both: i) the strict homology criteria we applied and ii) the sparse nature of representative sequences for domain families that have broad functionalities and potentially diverse evolutionary trajectories. Both *AIID* and *Ldh_2* belong to the Rossmann-fold dehydrogenase superfamily, but they are distinguished by their differing substrate specificities, where MLTH is more aligned with the *Ldh_2* family since this family primarily binds hydroxyorganic acids, such as malate and lactate. When compared to proteins of the reviewed function in these families (Figure 2B, Supplemental Figure 2), the putative MLTHs are more distant while maintaining a robust monophyletic clade, signifying potential evolutionary divergence.

Notably, MLTH exhibits an evolutionary relationship with Rossmann-fold dehydrogenase families despite lacking the canonical Rossmann signature motif and not utilizing mobile electron carriers as cofactors. Instead, earlier biochemical studies demonstrated that MLTH contains a covalently bound NAD⁺/NADH cofactor and catalyzes its reaction via a ping–pong mechanism.^12^ Although these findings distinguish MLTH from classical Rossmann-fold enzymes, the molecular basis underlying its catalytic activity remains unresolved. Structural characterization of MLTH will, therefore, be critical not only for elucidating its catalytic mechanism but also for providing deeper insight into the evolutionary adaptations that gave rise to this atypical member of the dehydrogenase superfamily. These future studies would also have wider implications given the growing body of research that highlights the increasing importance of *V. parvula* on human health.

Beyond its established role as a key member of the human oral microbiota, *V. parvula* is increasingly recognized as an important species in other medical contexts. For example, it has been implicated in inflammatory bowel disease, where it colonizes the inflamed intestine.^27^ Intriguingly, *V. parvula* was also recently shown to reduce cancerous tumor growth in mice.^28^ Because tumor lactate levels are positively associated with tumor progression and metastasis, the ability of *V. parvula* to consume lactate appears central to its antitumor effect: Kefayat et al. demonstrated that intravenous inoculation of *V. parvula* into breast tumor–bearing mice significantly reduced both tumor lactate levels and tumor burden, decreasing tumor volume and liver metastases by 44.3% and 51.6%, respectively, compared to controls. At the heart of these diverse roles lies *V. parvula*'s ability to catabolize L-lactate—a process critically dependent on MLTH.

## Conclusion

In this work, we purified the MLTH and identified its sequence. In addition, we also corroborated the previous work centered around this enzyme's characteristics, namely, reaction stoichiometry, subunit molecular weight, and amino acid composition. Furthermore, we identified putative MLTH homologs within the genus *Veillonella* and showed that they form distinct clades among enzymes with similar domains and functions.

## Supplementary Material

Supplemental Material.docx Supplemental Material.docx

## Data Availability

The script used to calculate and compare amino acid sequences is available at https://gitlab.com/mmp-uva/mlth_identification. The full peptide summary report of the MASCOT search results is also available in this repository along with the raw fasta Supplemental files 1, 2, 3, and 4 used to perform the phylogenetic analysis. All other data presented in this study will be made available upon request. If you are interested in obtaining the data, please contact the corresponding author, Filipe Branco dos Santos, at f.brancodossantos@uva.nl.
