# Supplementary material for: Vpar_1595 encodes malate–lactate transhydrogenase: The first step in lactate metabolism within the genus Veillonella
Source: Gut Microbes Rep. 2026 Mar 14;3(1):2641986. doi: 10.1080/29933935.2026.2641986 (PMC13034625; doi:10.1080/29933935.2026.2641986)
Supplement: Supplemental Material.docx [file KGMR_A_2641986_SM5265.docx]

Supplemental Material to:

***Vpar_1595* encodes malate-lactate transhydrogenase: the first step in lactate metabolism within the genus *Veillonella***

Maxwell Calvin Guillaume^1^, Filipe Branco dos Santos^1*^

^1^Swammerdam Institute for Life Sciences, University of Amsterdam, Amsterdam, The Netherlands

***Corresponding author:**

f.brancodossantos@uva.nl

**Supplemental Table 1: Top 5 candidate proteins from the *V. parvula* genome identified in the MASCOT search results.**

**
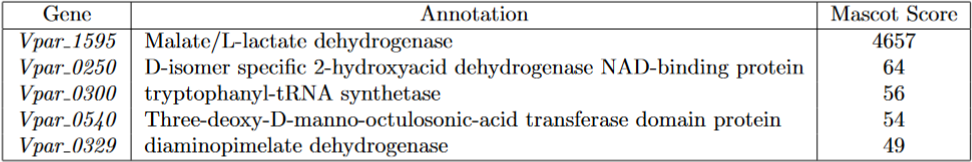
**

**Supplemental Table 2: Molecular Weight and AA composition comparison between measured MLTH characteristics in Allen *et al.* 1982 and calculated characteristics from primary structure.** The top three candidate proteins from the *V. parvula* genome are shown, as determined by the absolute total difference in percent amino acid composition when compared to MLTH from Allen and Patil*,* 1972. Note that amino acids asparagine (N) and glutamine (Q) are not included in the total difference calculation as they were not measured in Allen and Patil*,* 1972.

**
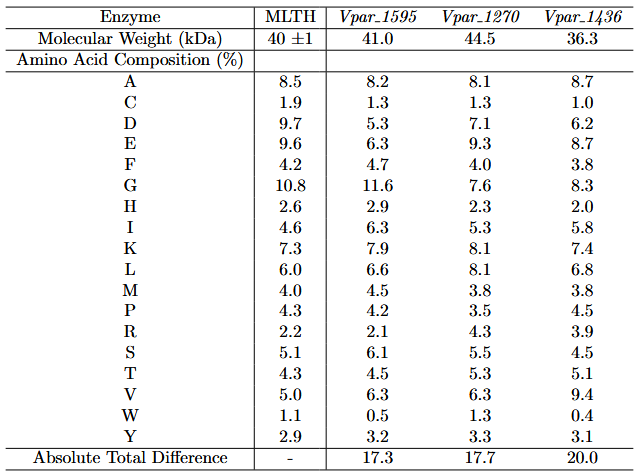
**

**Supplemental Table 3: The amino acid sequence of *Vpar_1595*.**

| Gene | *Vpar_1595* |
| --- | --- |
| Amino Acid Sequence | MADAKNTVLFPYETLKKLSMDAFQKFGFSEKEADIIQDVLLTSDLFGIQSHGMQRMVRYHKGITNGLIKIDAKPEIVKETPISAVIDGHDGMGQLLGHKAMEMAIEKAKKSGVGIVSVRNSNHYGIAGYYAKMASDQGLIGFSCTNSEAIMVPVYARKAMLGSNPIAWTVPADPVDFFFDCSTTVVTRGKLEMYNKMGKATPDGWAVNKDGVPSTDAAEVLGNISRHEGGGILPLGGATEVLGGHKGYGNGMIAELFSSILSQGGTSNKCMVGGKSNICHGFMAINPEFFGDPAEIKKHFSQFLQELREAPKAQGQDRIYTHGEKEHESVAKVKAEGIPVLNGTMLEVQDLCNELGLDFKSYFGDYVPEAPATMFKGNY |

**
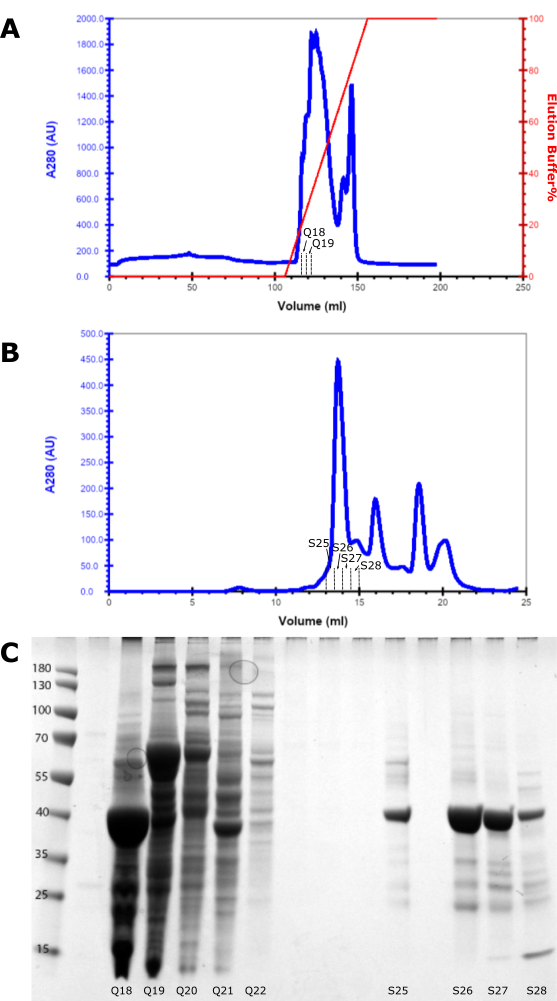
**

**Supplemental Figure 1: Purification of protein lysate from *V. parvula* to protein fractions with positive MLTH activity. A.** Ion exchange chromatograph of dialyzed cell lysate from *V. parvula* using a HiQTrap HP column. Blue axis signifies total protein content as measured by UV absorbance at 280 nm. The red axis shows the linear transition from loading buffer to elution buffer as percentage of elution buffer in the total eluent. The x axis is the total volume of eluent. Fractions with MLTH activity are labeled and delineated by the dashed black lines. **B.** Size exclusion chromatograph of concentrated fraction Q18 using a Superdex 200 column. **C.** SDS-PAGE gel of fractions with MLTH activity**.** Fractions are labeled at the bottom of the well. Note that fractions Q20-Q22 did not test positive for MLTH activity.


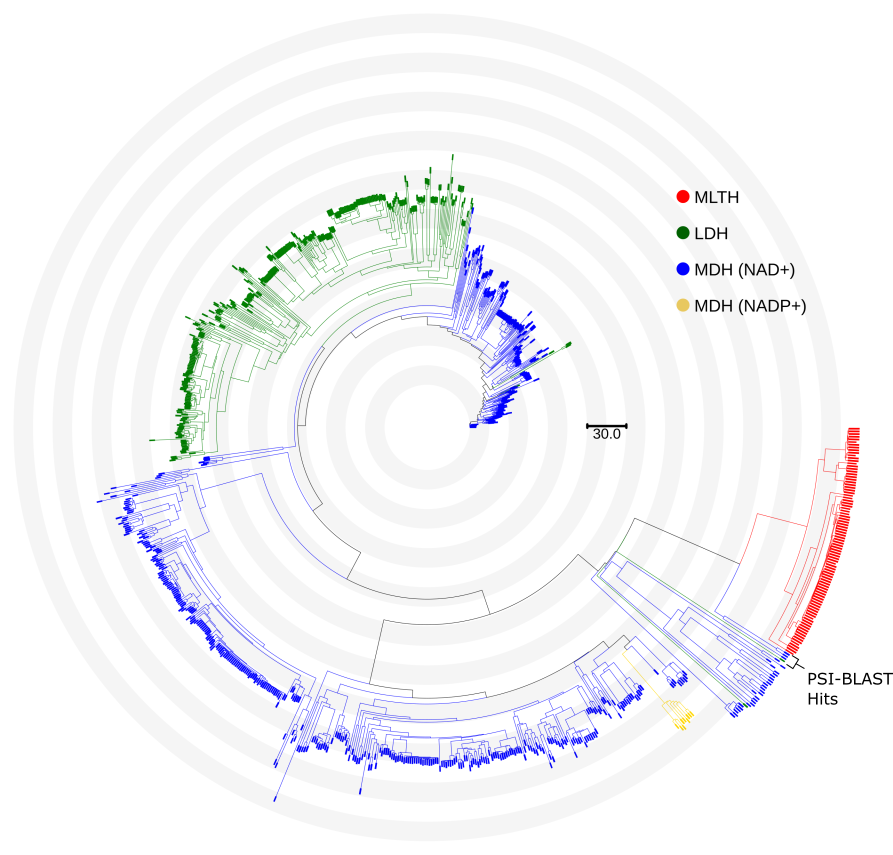


**Supplemental Figure 2: Phylogenetic tree of reviewed canonical LDH and MDH sequences and putative MLTH sequences.** LDH (EC:1.1.1.27), MDH (NAD+) (EC:1.1.1.37), and MDH (NADP+) (EC:1.1.1.82) sequences were obtained from Swissprot by searching for their corresponding EC numbers on Uniprot. Gray and white bands indicate a distance of 15, as indicated by the scale bar which traverses two bands. Note that the 4 nodes closest to the MLTH clade demarcated as PSI-BLAST Hits correspond to the three MDH nodes and one LDH node in **Figure 2B**.

The **Supplemental files** **1**, **2**, **3** and **4**, including the raw fasta sequences used during the phylogenetic analysis performed in this study, are available at <https://gitlab.com/mmp-uva/mlth_identification>.
